# Frank-ter Haar syndrome associated with sagittal craniosynostosis and raised intracranial pressure

**DOI:** 10.1186/1471-2350-13-104

**Published:** 2012-11-09

**Authors:** Charlotte L Bendon, Aimée L Fenwick, Jane A Hurst, Gudrun Nürnberg, Peter Nürnberg, Steven A Wall, Andrew OM Wilkie, David Johnson

**Affiliations:** 1 Oxford Craniofacial Unit, Oxford University Hospitals NHS Trust, John Radcliffe Hospital, Oxford, OX3 9DU, UK; 2 Weatherall Institute of Molecular Medicine, University of Oxford, Oxford, UK; 3 Department of Clinical Genetics, Oxford University Hospitals NHS Trust, John Radcliffe Hospital, Oxford, UK; 4 Cologne Center for Genomics, University of Cologne, Cologne, Germany; 5 Center for Molecular Medicine Cologne (CMMC), University of Cologne, Cologne, Germany; 6 Cologne Excellence Cluster on Cellular Stress Responses in Aging-Associated Diseases (CECAD), University of Cologne, Cologne, Germany; 7 Current address: Department of Clinical Genetics, Great Ormond Street Hospital NHS Foundation Trust, WC1N 3BH, UK

**Keywords:** Frank-ter Haar syndrome, Craniosynostosis, Sagittal synostosis, Intracranial pressure

## Abstract

**Background:**

Frank-ter Haar syndrome is a rare disorder associated with skeletal, cardiac, ocular and craniofacial features including hypertelorism and brachycephaly. The most common underlying genetic defect in Frank-ter Haar syndrome appears to be a mutation in the *SH3PXD2B* gene on chromosome 5q35.1. Craniosynostosis, or premature fusion of the calvarial sutures, has not previously been described in Frank-ter Haar syndrome.

**Case presentation:**

We present a family of three affected siblings born to consanguineous parents with clinical features in keeping with a diagnosis of Frank-ter Haar syndrome. All three siblings have a novel mutation caused by the deletion of exon 13 of the *SH3PXD2B* gene. Two of the three siblings also have non-scaphocephalic sagittal synostosis associated with raised intracranial pressure.

**Conclusion:**

The clinical features of craniosynostosis and raised intracranial pressure in this family with a confirmed diagnosis of Frank-ter Haar syndrome expand the clinical spectrum of the disease. The abnormal cranial proportions in a mouse model of the disease suggests that the association is not coincidental. The possibility of craniosynostosis should be considered in individuals with a suspected diagnosis of Frank-ter Haar syndrome.

## Background

Frank-ter Haar syndrome is a rare disorder comprising cardiovascular, skeletal and craniofacial anomalies including hypertelorism, brachycephaly and a wide anterior fontanelle [1]. The ‘ter Haar’ syndrome was initially described in three related children, all of whom developed severe cardiovascular complications [2,3]. These patients appeared to share many of the craniofacial and skeletal features normally associated with Melnick-Needles Syndrome, an X-linked congenital disorder of skeletal dysplasia. However the autosomal recessive pattern of inheritance and congenital cardiac defects distinguished the syndrome as a separate entity [4,5]. The condition affecting this subgroup of patients was renamed Frank-ter Haar syndrome following the realisation that ter Haar’s three patients shared many of the features described previously by Frank in an 18 month old female with megalocornea, skeletal dysplasia and developmental delay [1,6].

A genetic basis for Frank-ter Haar syndrome has recently been established through homozygosity mapping studies in patients from 12 affected families, identifying homozygous mutations in the *SH3PXD2B* gene on chromosome 5q35.1 as the most common cause [7]. The analysis of patients from 13 families identified 4 different intragenic mutations, and one complete deletion of *SH3PXD2B*, accounting for the phenotype in 7 of the families [7]. Clinical features characterising this group of mutation positive patients include brachydactyly megalocornea, hypertelorism, a prominent forehead, brachycephaly, a wide anterior fontanelle, micrognathia, a broad mouth and full cheeks [7]. *Sh3pxd2b* null mice appear to share many of the skeletal, craniofacial, cardiac and ocular defects described in Frank-ter Haar syndrome, supporting the link between this gene and the syndrome [7].

The craniofacial features of Frank-ter Haar syndrome are numerous however to our knowledge craniosynostosis has not been reported previously. Craniosynostosis refers to the premature fusion of one or more of the calvarial sutures, affecting 1 in 2500 individuals [8]. The calvarial sutures arise where cranial ossification centres meet at approximately 18 weeks of embryonic development. From this stage onwards further skull growth is appositional, and deposition of new mineralised bone matrix occurs along the margins of the sutures [8]. Synchronised closure of these sutures during the postnatal period allows the calvarium to achieve its full size and shape, facilitating normal expansion and development of the underlying brain [9]. Growth restriction along a given margin may result in compensatory, excessive growth at other sutures leading to skull deformity [10]. Furthermore as the brain continues to develop and expand within a limited cranial capacity, raised intracranial pressure (ICP) may complicate craniosynostosis, with potential neurodevelopmental consequences [11].

Here we describe three siblings with the Frank-ter Haar syndrome phenotype, all of whom are homozygous for a complete deletion of exon 13 of the *SH3PXD2B* gene. They demonstrate many of the classic features associated with Frank-ter Haar syndrome, however two of them also have presented with non-scaphocephalic sagittal synostosis complicated by raised ICP.

## Case presentation

**Figure 1:**
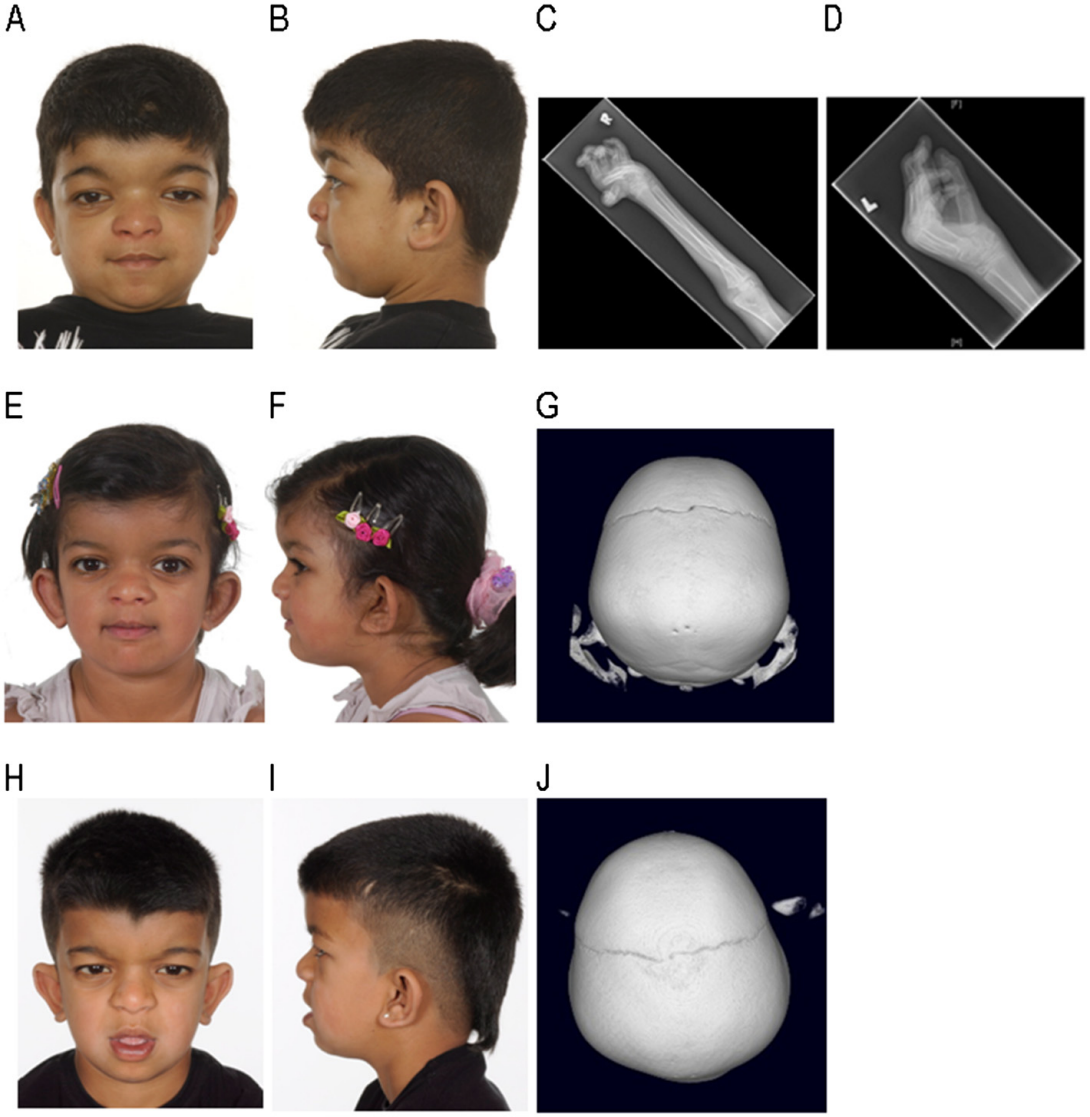
**A-D: Patient 1.A**: Antero-posterior (AP) view showing facial features including hypertelorism. **B**: Lateral view showing brachycephaly and micrognathia. **C**: X-ray (XR) right radius/ulna showing broad appearance of the radius at the junction between the proximal and middle thirds. **D**: XR left hand showing crowding of the carpal bones, broad metacarpals, proximal and middle phalanges, and flexion at the MCP and PIP joints. **E**-**G: Patient 2.E**: AP view showing facial features including hypertelorism. **F**: Lateral view showing brachycephaly. **G**: 3D CT scan showing absence of the sagittal suture. **H**-**J: Patient 3.H**: AP view showing facial features including hypertelorism. **I**: Lateral view showing class III malocclusion and brachycephaly. **J**: 3D CT scan showing absence of the sagittal suture.

We present a family of four children born to consanguineous (first cousin) parents originating from Kashmir, Pakistan. Three siblings (two males and one female) have Frank-ter Haar syndrome (Table 1, Figure 1) confirmed by genetic analysis. The youngest male sibling appears to be unaffected.

**Table 1:** **Comparison of clinical features in patients 1–3 with previous cases with prove *SH3PXD2B *mutations**

**Clinical features**	**Patient 1**	**Patient 2**	**Patient 3**	**Other 10 Frank-ter Haar cases with confirmed *SH3PXD2B *mutation **[7]
**General**				
Gender	M	F	M	M:F 8:2
Consanguinity	+	+	+	9/10
**Craniofacial**				
Prominent forehead	+	+	+	10/10
Hypertelorism	+	+	+	9/9
Brachycephaly	+	+	+	10/10
Wide anterior fontanelle	+	-	+	10/10
Prominent ears	+	+	+	5/7
Flat nasal bridge	+	+	+	
Micrognathia	+	-	-	9/10
Class III malocclusion	-	+	+	†
Anterior open bite	-	-	+	†
Open metopic suture	+	-	+	†
Sagittal synostosis	-	+	+	†
Raised intracranial pressure	-	+	+	†
Hypoplasia of teeth	+	+	+	
Broad mouth	+	+	+	10/10
Broad alveolar ridges	NR	NR	NR	6/8
Anteverted nostrils	-	-	-	6/9
Full cheeks	+	+	+	10/10
**Skeletal**				
Talipes	+	-	+	5/8
Size discrepancy in feet	-	+	-	†
Exostoses	+	+	-	†
Subcutaneous nodules	+	+	-	†
Contractures/flexion deformity fingers/clawing	+	-	-	3/10
Short hands/digits, brachydactyly	+	+	+	10/10
Kyphosis	-	-	+	5/8
Bowing of long bones	NT	NT	NT	7/10
Prominent coccyx	+	-	-	8/10
**Cardiac**				
Aortic regurgitation/prolapse	AVR	AVR	-	1/6
Tricuspid regurgitation	+	-	-	
Mitral valve prolapse/regurgitation	MVP/MVR	MVP/MVR	MVR	3/6
Ventricular septal defect	-	-	-	5/7
Double right outlet	-	-	-	2/3
**Ocular**				
Megalocornea	-	-	-	9/9
Congenital glaucoma/raised IOP	-	-	-	
**Other**				
Recurrent UTIs/duplex system	NT	+	NT	
Bilateral lymphoedema legs	+	-	-	†

Comparison is made with features in other previously-reported confirmed cases of Frank-ter Haar syndrome with known *SH3PXD2B* mutations [7].

† − Features not described in any previous reports or Frank-ter Haar syndrome, regardless of whether or not genetic analysis was performed to confirm the diagnosis [1-3,5,7,12-15].

NR - not recorded.

NT - not tested.

AVR - aortic valve regurgitation.

MVR - mitral valve regurgitation.

MVP - mitral valve prolapse.

### Patient 1

This male patient was the firstborn child, delivered by caesarean section for macrocephaly and cephalopelvic disproportion. Hypertelorism and dysmorphia were noted at birth, without evidence of fetal head constraint. He was referred to the Plastic Surgery Department for severe hypertelorism and forehead bossing. On examination at 20 months the head circumference was 49 cm (>99.6^th^ centile), with marked hypertelorism, frontal skull bossing, a wide open anterior fontanelle, and a persistent metopic suture. The cephalic index was 86% confirming the clinical appearance of mild brachycephaly (normal range 76-85%). Limb examination revealed severe bilateral cavo varus of the feet and bilateral metacarpo-phalangeal (MCP) joint hyperextension and contractures.

CT head and 3D studies at age 1 year and 10 months demonstrated patency of the major cranial sutures and confirmed the clinical findings of hypertelorism and an open anterior fontanelle. Surgical correction for bilateral cavo varus at age 7 years and 4 months was complicated by cardiopulmonary decompensation, pulmonary hypertension and a marked oxygen requirement during the post-operative period. Echocardiography demonstrated severe mitral valve prolapse with moderate mitral regurgitation, mild tricuspid valve prolapse and mild aortic regurgitation, however ventricular function was good at the time.

**Figure 2:**
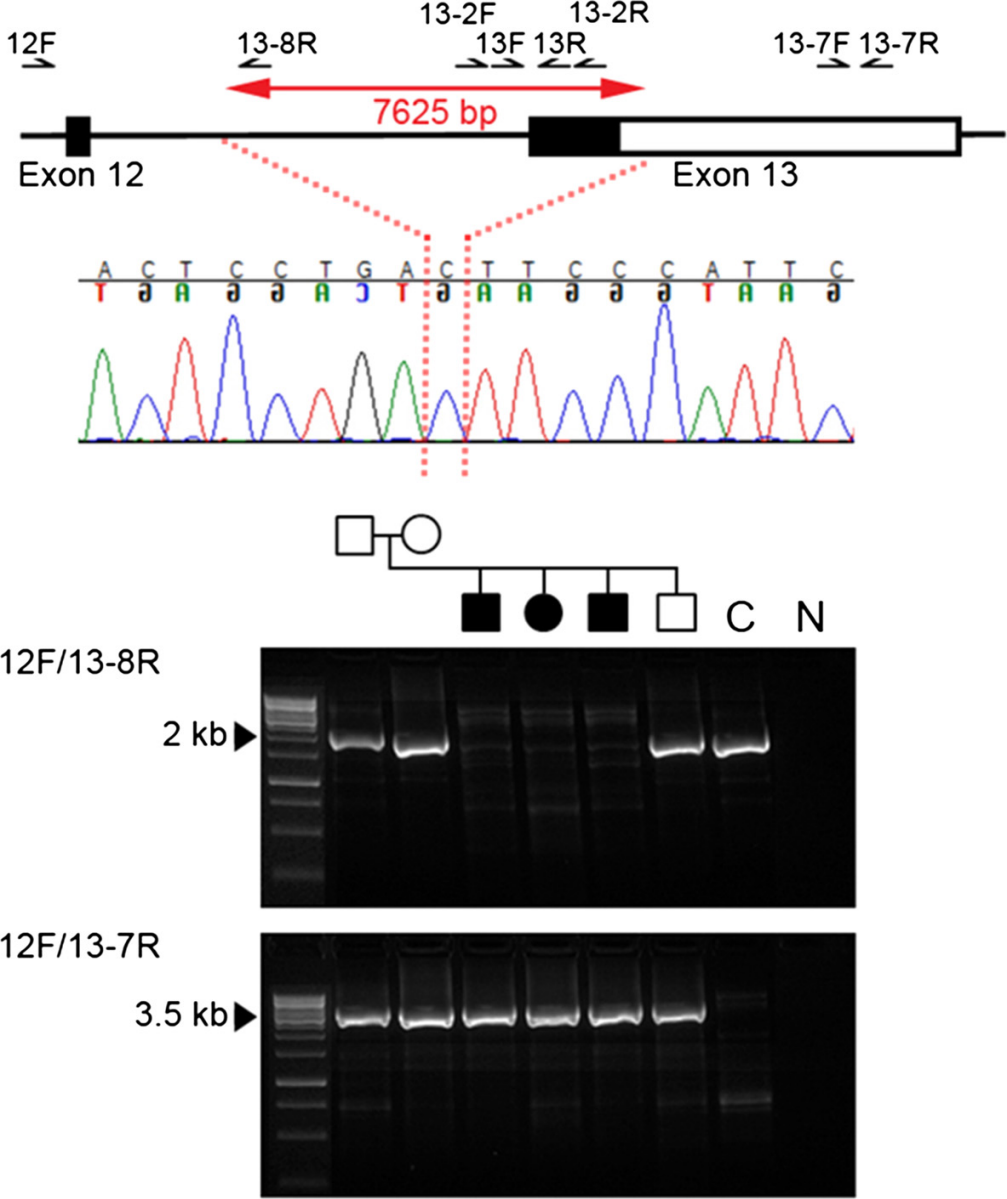
**Genome and sequence context of deletion in *SH3PXD2B. ***The upper panel shows a schematic representation (not to scale) of *SH3PXD2B* around the deleted region. Exons are shown as rectangles with coding sequence filled black and the 3^′^ UTR unfilled. Affected individuals were homozygous for a 7,625 bp deletion (double-headed red arrow). The breakpoint (dotted red lines) occurred at the position shown in the sequence chromatogram from patient 2, with an ambiguity of one nucleotide because a cytosine is located at both breakpoints. The lower panel shows the results of PCR with the primer pairs indicated. Individuals homozygous for the deletion failed to amplify using primer pair 12F/13-8R (upper gel image, 2,268 bp product); all family members yielded a truncated product using primer pair 12F/13-7R (lower gel image; non-deleted product would be 11,464 bp) indicating that unaffected family members are heterozygous for the deletion. C: control DNA from an unaffected, unrelated individual. N: negative control. The hg19 co-ordinates of the deleted region are chr5:171,763,754-171,771,378.

**Figure 3:**
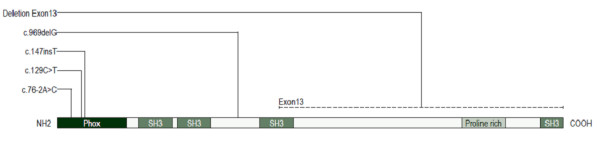
**Schematic plan of *SH3PXD2B *(TKS4) protein showing all mutations identified to date and their location in relation to domains defined by PROSITE (prosite.expasy.org).** Note the extent of the deletion identified in this family. A deletion of the entire encoding *SH3PXD2B* gene has also been reported in another affected family [7].

The diagnosis of Frank-ter Haar syndrome was initially suspected at the age of 18 months. The G-banded karyotype was normal (46,XY). Alternative clinical diagnoses considered included Winchester syndrome and geleophysic dysplasia, but genetic testing for these disorders was normal. The diagnosis was confirmed following the identification of the *SH3PXD2B* mutation at age 12 years and 4 months (Figure 2, Figure 3).

The mitral valve function subsequently deteriorated resulting in ventricular dilatation and requiring mitral valve repair at 12 years and 5 months. Examination at age 12 years and 11 months revealed a progressive lower limb oedema, which had been developing over a 2 year period, and was initially suspected to be a complication of his cardiac condition. Originally affecting the right leg only, it had progressed to involve the left groin, left leg and genitalia, and appeared to be lymphatic in origin. Lymphoscintigraphy studies at age 13 years and 10 months demonstrated profound lymphatic abnormalities in both lower legs and functional hypoplasia of the main draining lymphatics.

This patient has a left-sided astigmatism and meibomian gland dysfunction; however he does not have any of the ocular complications classically associated with Frank-ter Haar syndrome, such as megalocornea and glaucoma [7,16]. Intraocular pressures have been within the normal range, without clinical evidence of glaucoma. He has been attending a mainstream school with learning support, and is under occupational therapy for difficulties with fine motor skills, washing and dressing as a result of the severe hand contractures and deformity.

### Patient 2

This is the second affected sibling in the family, delivered by caesarean section for maternal proteinuria without evidence of fetal head constraint. Facial features including hypertelorism were noticed at birth. Examination on presentation to the craniofacial department at age 6 years and 6 months demonstrated hypertelorism, brachycephaly (cephalic index 89%), a flat nasal bridge, prominent ears, a class III malocclusion and hypoplastic teeth. Her fingers and toes appeared shortened and exostoses on the dorsal aspect of the radius and ulna were visible on examination of the arms.

CT head and 3D studies were carried out at age 6 years and 10 months to further delineate the craniofacial features. This demonstrated sagittal synostosis, however the skull was not scaphocephalic in shape, but instead appeared brachycephalic in keeping with the clinical examination findings. There was evidence of limited space within the supratentorial compartment, and small ventricles. Although neurological development had previously been within normal limits, difficulties with literacy and recurrent headaches were reported at this time, also suggestive of raised ICP. ICP monitoring (Codman Microsensor, Codman and Shurtleff Inc., Raynham, Mass.) demonstrated elevated pressures with a mean of 22 mmHg and 5 significant associated B-waves. Calvarial expansion was successfully undertaken. She made a good post-operative recovery, and the headaches subsequently resolved.

This sibling has also developed cardiac complications but has been less severely affected than her older sibling. Echocardiography at age 7 years and 6 months demonstrated mild-moderate mitral regurgitation with mitral valve prolapse, and minimal aortic regurgitation. Left ventricular function is currently stable. The diagnosis of Frank-ter Haar syndrome was made at age 7 years and 7 months following genetic analysis of the family (Figure 2).

This patient has not developed any of the ocular complications classically associated with Frank-ter Haar syndrome and intraocular pressures and appearance of the retina have been within normal limits. She has been attending a mainstream school, but has experienced some difficulties with literacy.

### Patient 3

Patient 3 is the younger affected male sibling. He was delivered by caesarean section following a normal pregnancy, without evidence of fetal head constraint. Facial features in keeping with Frank-ter Haar syndrome were noticed soon after birth including hypertelorism, a broad forehead, wide anterior fontanelle, brachycephaly and prominent ear*s*. The diagnosis of Frank-ter Haar syndrome was made at age 6 years and 5 months, following genetic analysis of the family (Figure 2).

CT head and 3D studies were performed at age 6 years and 9 months following the outcome of these investigations in Patient 2. Evidence of sagittal synostosis without scaphocephaly, a flattened occiput and shortened AP diameter were identified on CT. There was hypoplasia of the inferior vermis but an otherwise normal appearance of the brain. Given the history of raised ICP in his sibling, ICP monitoring was undertaken at age 6 years and 10 months, demonstrating a mean pressure of 10 mmHg but 7 B waves, indicating raised ICP.

Examination at age 7 years and 1 month demonstrated an anterior open bite, class III malocclusion, hypoplasic teeth and skeletal features including short fingers and toes, and a mild positional talipes. The cephalic index was 88% in keeping with the clinical appearance of brachycephaly. Calvarial expansion was performed at age 7 years and 7 months.

Cardiac complications have also been documented, but have been less severe than the other affected siblings, with evidence of mild mitral regurgitation on echocardiography. No ocular complications have been reported, and intraocular pressures have been within the normal range. He has required some learning support for literacy and numeracy, but developmental progress and performance at school have been within normal limits.

### Genetic analysis

We genotyped DNA samples from three affected patients, one unaffected sibling and their parents using the Affymetrix GeneChip® Human Mapping 250K Sty Array. Linkage analysis was performed assuming autosomal recessive inheritance, full penetrance, consanguinity and a disease allele frequency of 0.0001. Multipoint LOD scores were calculated using the program ALLEGRO [17]. We identified three regions of shared homozygosity on chromosomes 5 (150943995–173861414; hg18 coordinates), 8 (134041593–146264218) and 12 (115552170–123960781). Following the identification of mutations in Frank-ter Haar syndrome [7], it was noted that the region of homozygosity on chromosome 5 included the *SH3PXD2B* gene. All coding exons of *SH3PXD2B* were therefore PCR amplified in samples from the family (primer sequences available on request). As exon 13 failed to amplify (using primers 13F, 5^′^-AACATCTCCATTGGTGGTCC-3^′^ and 13R, 5^′^-GATGTGTTTGGCTGGCATC-3^′^, positions of primers are shown in Figure 2) in the affected individuals, an alternative primer pair (13-2F, 5^′^-CATGTAAGATATTCCCGGAACATGGT-3^′^; 13-2R, 5^′^-CCCCATGTCATTTTTCAGCTGGAACA-3^′^) was designed to rule out the possibility of a SNP underlying the primer and preventing annealing. This also failed to amplify in the 3 affected individuals, confirming a homozygous deletion of exon 13 of *SH3PXD2B*. Further primer pairs were designed throughout the 3^′^ untranslated region (UTR) to identify the extent of the deletion (primer sequences available on request), a primer pair near the end of the 3^′^ UTR successfully amplified in the affected individuals (13-7F, 5^′^-TACCTTACATCCCAGGGCAAACGGACAGCT-3^′^; 13-7R, 5^′^-GCGTGGCCTTGTGGCAGAGGTTTAAAATGAC-3^′^), the reverse primer of which was then used in a long PCR with the 12F primer (5^′^-AATCCAACTAGGTCCCCAGC-3^′^). This yielded a truncated product of ~3.5 kb rather than the normal 11,464 kb, which was sequenced to identify the exact breakpoint. All family members produced this smaller PCR product, demonstrating that unaffected members are heterozygous carriers of the allele bearing the deletion, which preferentially amplifies during PCR. The non-deleted allele was demonstrated by PCR between 12F and 13-8R (5^′^-CATAACACATCCTGAAGATAAACAGCCTAGACA-3^′^). The complete deletion of the coding part of exon 13 is predicted to abolish over half of the protein, including the SH3 domains and a proline-rich domain (Figure 3).

## Conclusions

Frank-ter Haar syndrome is a rare, autosomal recessive disorder. We present three siblings born to consanguineous parents, who are homozygous for a deletion of exon 13 of the *SH3PXD2B* gene. These three children share many of the characteristic physical features of the Frank-ter Haar phenotype, however two of the three siblings also developed non-scaphocephalic sagittal synostosis, both cases being complicated by raised ICP. Craniosynostosis and raised ICP have not been described in previous reports of Frank ter-Haar syndrome.

The sagittal suture is involved in 50-60% of cases of craniosynostosis, and is therefore the most common suture to be synostosed [8,10]. Familial or genetic factors are less significant in sagittal synostosis than in other forms of craniosynostosis. In a large study of over 500 patients with isolated sagittal synostosis familial factors were identified in only 6% [18]. Most such cases are likely to be multifactorial, as causative genetic mutations are only rarely identified [19-21]. In comparison genetic factors comprising chromosomal abnormalities and gene mutations appear to account for at least 21% of cases of craniosynostosis overall [22]. Intrauterine fetal head constraint has been implicated as an important causative factor in sagittal synostosis [23], however to our knowledge neither of our two Frank-ter Haar siblings with sagittal synostosis had a history of intrauterine fetal head constraint.

It is widely acknowledged that premature fusion of the calvarial sutures may restrict normal growth and development of the underlying brain especially in syndromic cases. This can be complicated by raised ICP [10]. Raised ICP may be defined as an elevated baseline ≥15 mmHg, or >3 B waves (levels of 20–50 mmHg lasting 5–10 minutes before returning to baseline) in a 24 hour period [24,25]. In early studies, raised ICP was demonstrated in only 14% of cases of single suture synostosis but affected almost 50% of cases involving more than one suture [11]. The discovery of raised ICP in both of our patients with Frank-ter Haar syndrome and sagittal synostosis is unusual. Scaphocephaly is a predictable consequence of sagittal synostosis and refers to a boat-shaped skull deformity, with an increased anteroposterior diameter, decreased biparietal diameter and reduced cephalic index (<76%) In contrast, non-scaphocephalic sagittal synostosis is a rare diagnosis [25]. In the first reported cases of non-scaphocephalic sagittal synostosis only eight patients were identified out of 193 patients with isolated sagittal synostosis [25]. It is interesting to note that a diagnosis of raised ICP was made in 66% of these non-scaphocephalic cases, much higher than the reported incidence of 14% for all single suture synostosis [11].

An underlying genetic defect has recently been identified in Frank-ter Haar syndrome following homozygosity mapping in 16 patients from 12 affected families [7]. The mutated gene, *SH3PXD2B* on chromosome 5q35.1, encodes tyrosine kinase substrate with four Src homology 3 [SH3] domains (TKS4), a protein considered integral to the formation and functioning of podosomes. These are cell membrane protrusions which facilitate cell adhesion, migration, and extracellular matrix degradation [7]. Two mouse models with *Sh3pxd2b* loss of function mutations have been described. The first is a spontaneous *nee* mutation resulting from the deletion of a single base pair in exon 13 of the *Sh3pxd2b* gene (1303delA), which is thought to encode a truncated protein lacking part of the third and all of the fourth SH3 domains [16,26]. The second is an engineered null mutation created by the insertion of a gene trap vector between exons 3 and 4 of the *Sh3pxd2b* gene, which appears to result in a complete loss of gene expression [7]. Mice with the engineered null mutation appear to share many of the craniofacial, skeletal and cardiac features described in patients with Frank-ter Haar syndrome including mitral valve defects, hypertelorism and micrognathia [7,26,27]. Significantly, abnormal cranial proportions were described in these *Sh3pxd2b* null mice, which had a short snout, brachycephaly and hypertelorism, associated with persistently open sagittal sutures [7]. Both *Sh3pxd2b* null and Sh3pxd2b *nee* mice appear to develop ocular features including corneal opacities and anterior segment dysgenesis resulting in early-onset glaucoma. *Sh3pxd2b* nee mice also have abnormal craniofacial features including a short snout, brachycephalic skull, and Eustachian tube dysmorphology [16,26,27]. Surprisingly the ocular features associated with Frank-ter Haar syndrome were not identified in any of the three affected siblings, which contrasts both with the occurrence of megalcornea in all previous patients with identified *SH3PXD2B* mutations and with the ocular abnormalities described in both mouse models.

A variety of specific *SH3PXD2B* gene mutations have been reported in families with the Frank-ter Haar syndrome phenotype, but none so far have reported specific involvement of exon 13, the region of the gene affected in this family (Figure 3). However deletion of the entire *SH3PXD2B* gene has been described in one family [7]. Our three children have many features classically associated with Frank-ter Haar syndrome however they also have additional features which to our knowledge have not been described in previous reports (Table 1). These include lymphoedema, class III malocclusion, anterior open bite, open metopic suture, and non-scaphocephalic sagittal synostosis. Although we cannot completely exclude the possibility that these novel phenotypes are related to the two other regions of shared homozygosity identified on chromosomes 8 and 12 in this family, the involvement of the sagittal suture and additional craniofacial features in mouse models of the disease provides a precedent. The clinical features of craniosynostosis and raised ICP in this family therefore expand the clinical spectrum of Frank-ter Haar syndrome.

## Consent

Written informed consent was obtained from the family for publication of this case report and any accompanying images. A copy of the written consent is available for review by the Series Editor of this journal.

## Competing interests

The authors declare that they have no competing interests.

## Authors’ contributions

CLB collected the clinical data and wrote the paper. ALF performed the mutation analysis. JAH made the clinical diagnosis in the patients. GN and PN performed the homozygosity mapping. SAW supervised the clinical study. AOMW supervised the genetic studies and wrote the paper. DJ supervised the clinical study and wrote the paper. All authors read and approved the final manuscript.

## Pre-publication history

The pre-publication history for this paper can be accessed here:

http://www.biomedcentral.com/1471-2350/13/104/prepub
